# Traumatic Pseudoaneurysm of the Internal Maxillary Artery: A Rare Life-Threatening Hemorrhage as a Complication of Maxillofacial Fractures

**DOI:** 10.1155/2016/9168429

**Published:** 2016-11-23

**Authors:** E. Nastro Siniscalchi, L. Catalfamo, A. Pitrone, R. Papa, F. Famà, G. Lo Giudice, G. Cervino, M. Cicciu, F. S. De Ponte

**Affiliations:** ^1^Department of Clinical and Experimental Medicine Odontoiatric and Biomorfological Images, University of Messina, Messina, Italy; ^2^Department of Human Pathology, University of Messina, Messina, Italy

## Abstract

Pseudoaneurysm of the internal maxillary artery due to a traumatic event is a rare condition. Pseudoaneurysms are usually directly produced by arteries break with extravasation of blood. The compressed perivascular tissue forms the wall of aneurysmal sac. Then, this sac gradually expands and can be damaged. It is rare to see pseudoaneurysms of IMA. They are usually associated with fracture of the neck of the mandible. To the best of our knowledge the pseudoaneurysm of the internal maxillary artery related to maxillofacial trauma is an event extremely rare in the literature and if not quickly managed can lead to the patient's death. This case underlines how the close cooperation between surgeons and radiologists results in a quick diagnosis and management of such pathological events.

## 1. Introduction

Pseudoaneurysm (PA) is a rare life-threatening complication that consists of an incomplete tear of the vessel causing a blood flow into the surrounding tissues. If the inelasticity of the surrounding tissues allows a compressive effect, bleeding can be counterbalanced by this compressive action, leading to a formation of a hematoma [1–3].

Pseudoaneurysm has been reported as a consequence of mandibular fractures in the treatment of sagittal split ramus osteotomy, Le Fort I osteotomy, temporomandibular joint surgery, distraction osteogenesis, and trauma [1–6].

The internal maxillary artery (IMA) is the last terminal branch of the carotid artery. Because of its deep lie, hemorrhage can not be easily managed by digital pressure [7].

Treatment of such complication can be achieved by interventional radiology through a selective embolization of the vessel, which allows a well-acknowledged management with excellent outcomes. A case of early traumatic pseudoaneurysm of IMA as a consequence of maxillary and mandibular trauma and the treatment with endovascular embolization prior to open reduction and fixation of fractures is reported.

## 2. Case Report

**Figure 1:**
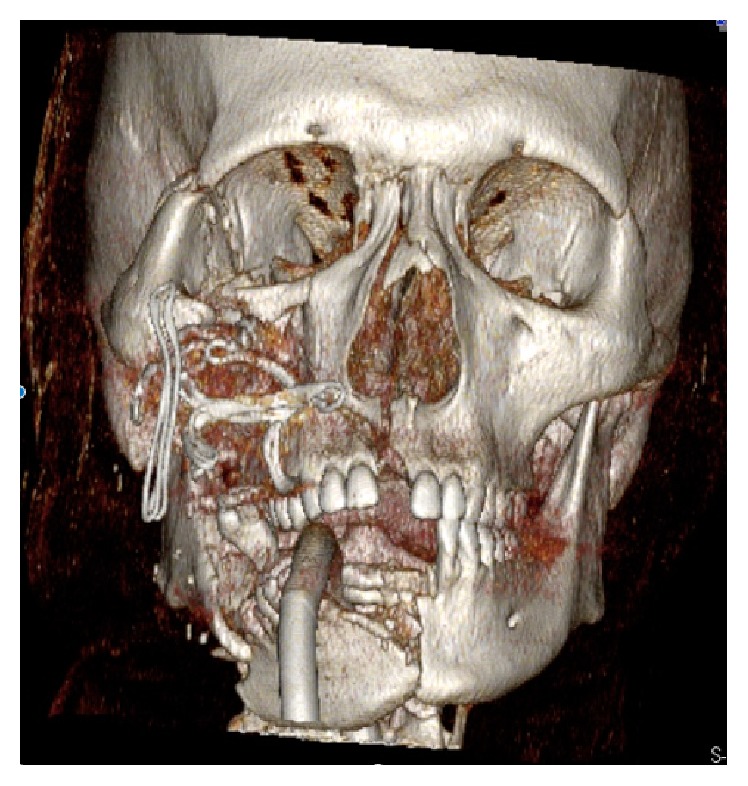
Three-dimensional reconstruction of the skull underlines the multiple facial fractures.

**Figure 2:**
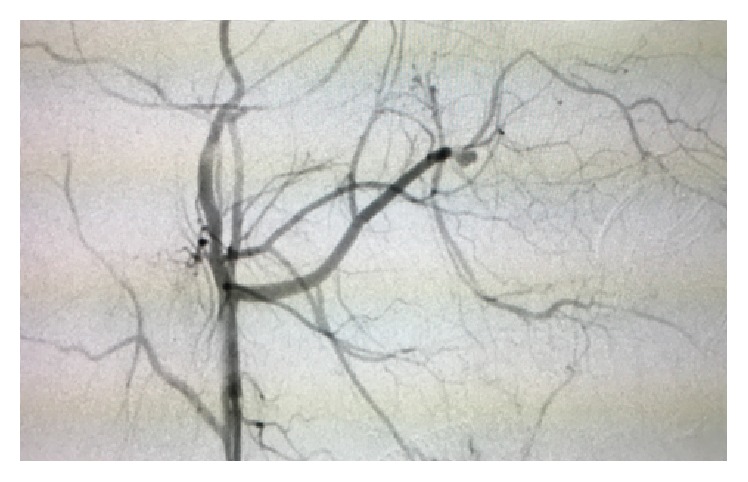
The diagnostic catheter evidences a dissecting posttraumatic pseudoaneurysm of the medium-distal portion of the internal maxillary artery.

**Figure 3:**
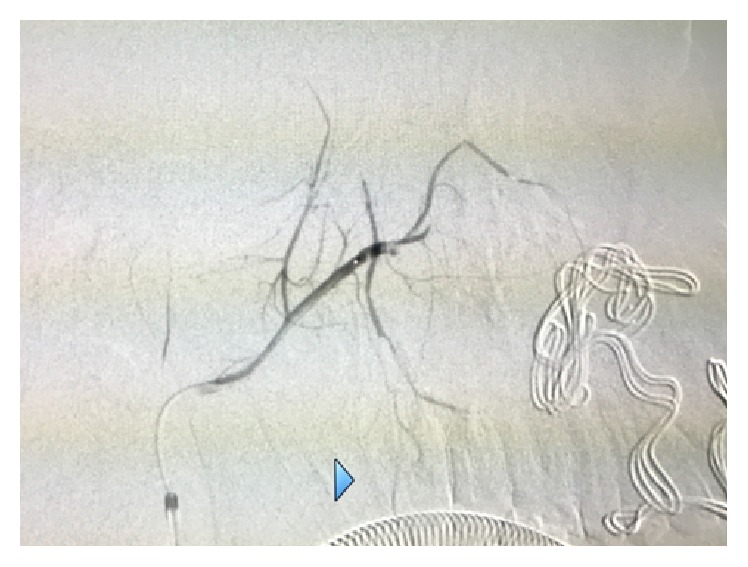
A microcatheter (EV3 Marathon) was navigated coaxially into the parent artery over a guidewire (EV3 Silver Speed) to reach the pseudoaneurysm.

**Figure 4:**
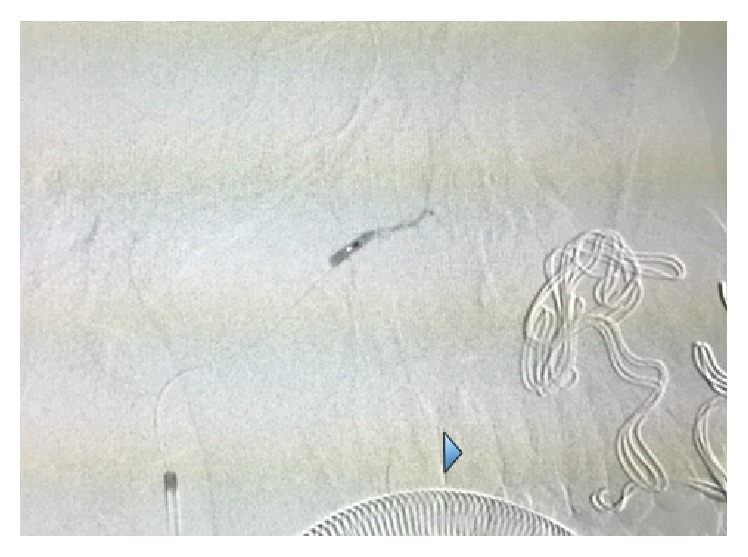
A mixture of 50% of acrylic-glue (Glubran 2) and Lipiodol ultrafluid was injected up to the complete occlusion of the lesion.

**Figure 5:**
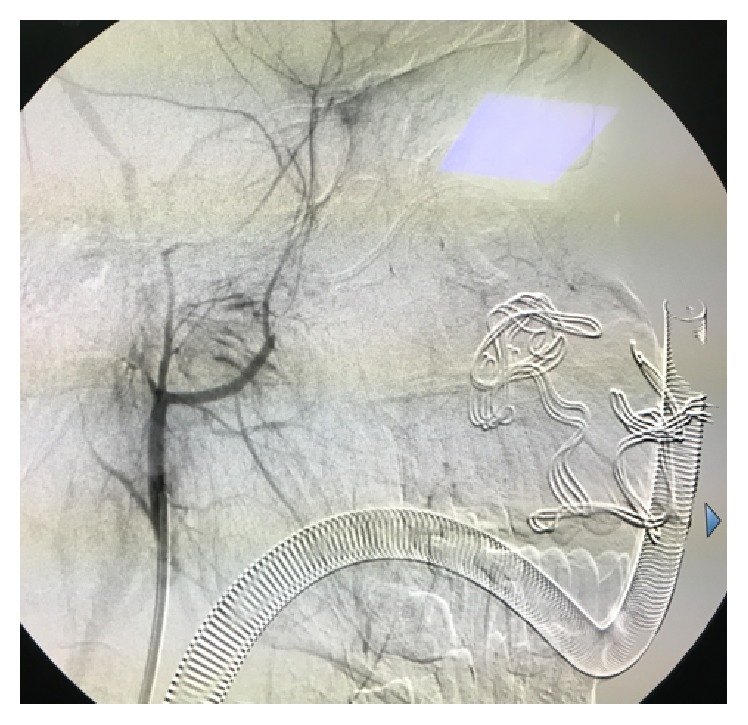
The complete exclusion of the pathological portion of the branch with the pseudoaneurysm is documented.

A 16-year-old Asian male was brought to the Maxillofacial Department of University Hospital of Messina. He was previously admitted at the Emergency Department of another hospital after a motorbike accident and immediately transferred to our Unit of Maxillofacial Trauma with a diagnosis of Le Fort III fracture and mandibular fracture associated with active bleeding of the right buccal mucosa. He has the pulse of 72/min and blood pressure (BP) of 112/70 mmHg with a normal FAST exam and presented 9,2 gr/dL of hemoglobin (Hb). The CT scan showed a mandibular right parasymphyseal fracture associated with complex maxillary fractures in a Le Fort III pattern (Figure 1). No condylar fractures were detected. Upon the admission to our department, his Glasgow Coma Scale (GCS) was 13/15. The clinical examination revealed a mobility of the midline of the mandible and maxilla with diffuse swelling and an unusual profuse bleeding from a mucosal wound in the right upper molar region. The patient was brought to the operative room and monitored. After several attempts with local measures (packing and electrocautery), the bleeding still remained with a blood loss of approximately 500 mL. A further blood examination revealed 7,6 gr/dL Hb. The patient was then transfused with 3 units of fresh frozen plasma and 3 units of erythrocyte. Clinically no signs of neck swelling were recorded. No pulsations were detected through auscultation. Fluid therapy was done with 1,000 mL of lactated ringers solution and the patient was immediately sent to Radiology Unit. The CT scan with intravenous contrast showed a focal rounded hyperdensity in the right infratemporal region. The patient was then referred for interventional radiology to perform an angiography that represents the “gold standard” to study arterial lesions. Angiography was performed with the patient under conscious sedation. The Seldinger technique was used to catheterize the right common femoral artery with a 5F groin sheath. A 5F diagnostic catheter was placed into the right external carotid artery. The angiograms confirmed the presence of a dissecting posttraumatic pseudoaneurysm of the medium-distal portion of the internal maxillary artery (Figure 2). No signs of active bleeding were observed during the angiographic study. A microcatheter (EV3 Marathon) was navigated coaxially into the parent artery over a guidewire (EV3 Silver Speed) to reach the pseudoaneurysm (Figure 3). A mixture of 50% of acrylic-glue (Glubran 2) and Lipiodol ultrafluid was injected up to the complete occlusion of the lesion (Figure 4). The final controls documented the complete exclusion of the pathological portion of the branch with the pseudoaneurysm (Figure 5).

Five days after the endovascular procedure open surgery with RIF was done once hemoglobin value was established at 10 gr/dL. The patient was then dehospitalized ten days after with 12 r/dL of hemoglobin.

**Figure 6:**
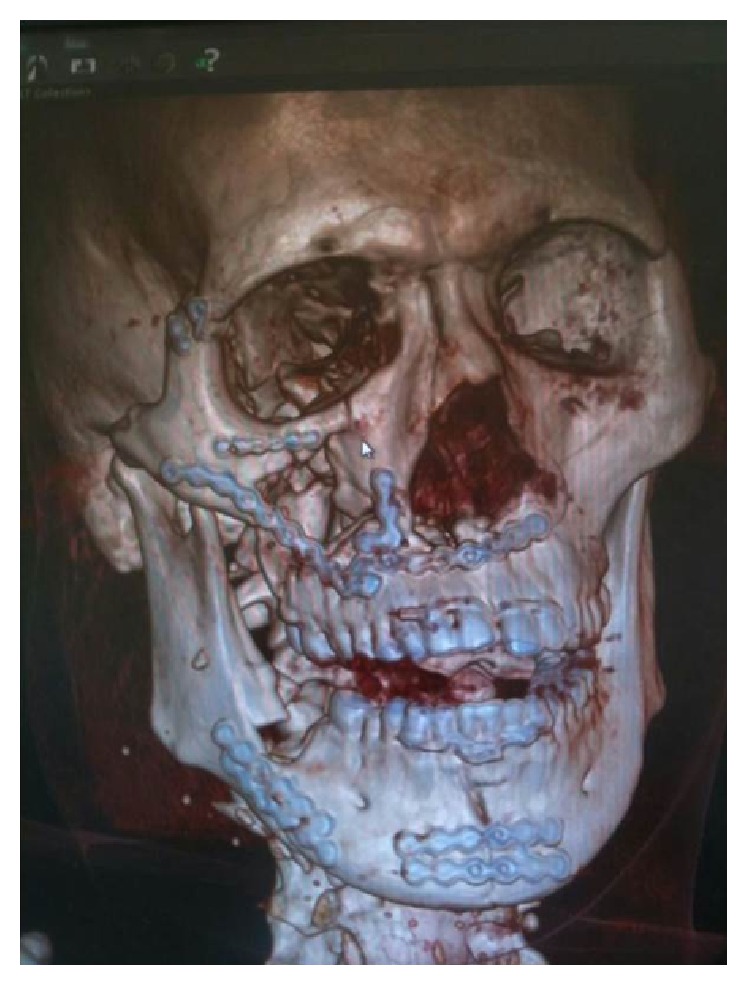
Postsurgical 3D CT scan.

The six-month clinical and radiological follow-up demonstrates good functional and aesthetic outcomes (Figure 6).

## 3. Discussion

Maxillary artery (MA) is the largest terminal branch of the external carotid artery. Because of its deep lie, hemorrhage can be difficult to manage [8–10]. It is classically divided into three portions, based on the relationship with the lateral pterygoid muscle: the mandibular portion, the pterygoid portion, and the pterygopalatine portion which is the deepest part of the artery. The mandibular portion is close to the mandibular condyle. Its mean distance from the neck of the condyle is approximately 6.8 mm. This close relationship can explain why lacerations of the artery can occur in this tract. Condylar fractures are one of the principle causes of MA lesions [10–12]. A possible mechanism of MA rupture in such tract is the direct lesion caused by fractured bone. This lesion can occur immediately during trauma or, in a second time, during subsequent motion of the fractured blocks.

Basing on the entity and the dynamic of the trauma, a lesion of MA can cause a classic hemorrhage, which is considered the most dangerous event. Pressure packing and topical hemostatic agents cannot be sufficient to arrest the bleeding because of the deep lie of the artery. Surgical ligation of the MA can be mandatory even if technically difficult. Ligation of external carotid artery can be considered as an extreme maneuver.

Pseudoaneurysm, or false aneurysm, is an uncommon consequence of arterial damage, resulting from an incomplete disruption of the arterial wall causing an expanding lesion between the artery and the surrounding tissues [11–14]. In such cases, the hematoma of the surrounding tissues counterbalances the arterial pressure, causing the hemorrhage, compressing and stabilizing the bleeding. This “natural package” limits the bleeding and if the tear is small lets the platelets form the clot and stabilize the bleeding with a consequent resolution of the hematoma. If the tear is bigger and platelets are not able to arrest the bleeding, a pseudoaneurysm can form. The PA is influenced by three factors: (1) the extent of the tera; (2) the elasticity of the surrounding tissues; and (3) the arterial blood flow [15]. Vascular tear depends on the dimension of the fractured bones, related to the artery. In the reported case, the maxillary bone fractured in a Le Fort III pattern could have caused the damage. In our experience, the early lesion of the artery could be formed with a sudden shift of the artery in a “concussion way.” This mechanism can explain those cases in which the entity of fractures seems not to be enough to cause a direct lesion of the artery. The inelasticity of the surrounding tissue of the MA can let the hematoma form a PA, above all in those tracts more compressed by dense connective tissues.

The diagnosis of PA, although rare, has to be suspected in every case of posttraumatic and postsurgical severe swelling of the face. Common etiology of PA of the IMA includes blunt and penetrating traumas, orthognathic surgery, neck dissection, surgical removal of impacted third molars, and radiotherapy [11, 14]. Contrast-enhanced CT and catheter angiography are the gold standard in diagnosing PA of the IMA [16].

Some cases of PA are reported to be spontaneously resolved. In other cases, complications like delayed hemorrhage, expansion, neuralgia, pain, and ischemia of the distal districts have been described [14, 16]. Because of the unpredictable course of the PA a treatment is requested once the correct diagnosis is made.

PA treatment includes various surgical and endovascular options. Surgical resection is not always possible due to accessing difficultly of deep-lying lesions [8, 11]. Besides, surgery increases the risk of damage to nerves and it could cause cosmetic defects as facial scars. Catheter-based embolization is a safe, quick, and effective technique and it avoids the morbidity of an extensive surgical exposure. Endovascular approach involves either the use of materials to occlude vessel lumen or the placement of a stent (covered or not) across the PA base [15]. The best treatment for PA originating from IMA is the occlusion of the affected artery, by transarterial embolic agents, distally at the level of the middle meningeal artery origin. In these cases the collateral circulation allows vessel sacrifice [16]. Numerous agents have been used for the embolization therapy such as metallic coils, polyvinyl alcohol particles, n-butyl cyanoacrylate (NBCA), polymers (Onyx, SQUID), and absorbable sponge gel [9, 11, 13]. Metallic coils are permanent embolic agent, with fibers attached or not. Coils are deposited into the vessel lumen proximal to the PA to arrest the flow; the positive charges of the titanium attract the negative charges of blood components, causing a thrombotic reaction to occlude the vessel. Fibers attached to the coils increase the thrombotic effect. The coils choice is fundamental because suitable size and length ensure adequate thrombosis and flow arrest, preventing the occlusion of normal vessels. NBCA, Onyx, and SQUID penetrate deeper into the vessels and they may go into the venous system. Takeshita et al. analyse some series on traumatic IMA PA treated with endovascular therapy [14]. They reported that the most common embolization agents used are metallic coils (40%), particles (28%), and NBCA (24%). They reported that NBCA was the most appropriate embolic material for the PA because embolization is completed more quickly compared to other agents, the primary hemostasis rate is higher, and the recurrent hemorrhage rate is lower [14–16]. However operators should be familiar with the use of NBCA because the reflux of polymerized glue around the microcatheter may adhere to its tip, increasing the risk of nontarget embolization or catheter retention. Parent artery occlusion with metallic coils is considered an effective procedure. A disadvantage of such procedure is the risk of recurrent hemorrhage due to retrograde filling of the PA through indirect collateral circulation. The filling of PA sac with coils may rupture the aneurysm wall, causing the migration of coils outside of the target lesion. Acute complications of endovascular treatment include distal thromboembolic events (occlusion of the central retinal artery, ischemic stroke due to potential anastomosis between the IMA and the ophthalmic artery) and local tissue infarct. Other reported complications are perforations, glued vein, microcatheter fracture, and vessel dissection or branch occlusion [13–16]. Moreover, to reduce the rate of complications, as blindness, facial palsy, and other cranial nerve palsies, it is mandatory to know the anastomosis between the external carotid artery and the internal carotid artery [15, 16].
